# Raman and Infrared spectroscopies and X-ray diffraction data on bupivacaine and ropivacaine complexed with 2-hydroxypropyl−β−cyclodextrin

**DOI:** 10.1016/j.dib.2017.08.053

**Published:** 2017-09-04

**Authors:** Murillo L. Martins, Juergen Eckert, Henrik Jacobsen, Everton C. dos Santos, Rosanna Ignazzi, Daniele Ribeiro de Araujo, Marie-Claire Bellissent-Funel, Francesca Natali, Michael Marek Koza, Aleksander Matic, Eneida de Paula, Heloisa N. Bordallo

**Affiliations:** a Niels Bohr Institute, University of Copenhagen, Universitetsparken 5 DK-2100, Copenhagen, Denmark; b Department of Chemistry, University of South Florida, 4202 E. Fowler Ave., Tampa, FL 33620, United States; c Theoretical Division, Los Alamos National Laboratory, Los Alamos, NM 87545, United States; d Department of Physics, Norwegian University of Science and Technology (NTNU), Høgskoleringen 5 NO-7491, Trondheim, Norway; e Human and Natural Sciences Center Federal University of ABC (UFABC), 09210-170 Santo André, SP, Brazil; f LLB, CEA, CNRS, Université Paris-Saclay, CEA Saclay, 91191 Gif-sur-Yvette, France; g Institute of Materials, Research National Council (CNR-IOM), Italy; h Institut Laue-Langevin, BP 156, F-38042 Grenoble Cedex 9, France; i Department of Applied Physics, Chalmers University of Technology, SE-41296 Göteborg, Sweden; j Department of Biochemistry and Tissue Biology, State University of Campinas (UNICAMP), 13083-862 Campinas, SP, Brazil; k European Spallation Source ESS AB, P.O. Box 176, SE-22100 Lund, Sweden

## Abstract

The data presented in this article are related to the research article entitled “Probing the dynamics of complexed local anesthetics via neutron scattering spectroscopy and DFT calculations (http://dx.doi.org/10.1016/j.ijpharm.2017.03.051)” (Martins et al., 2017) [1]. This work shows the molecular and structural behavior of the local anesthetics (LAs) bupivacaine (BVC, C_18_H_28_N_2_O) and ropivacaine (RVC, C_17_H_26_N_2_O) before and after complexation with the water-soluble oligosaccharide 2-hydroxypropyl−β−cyclodextrin (HP-β-CD).

**Specifications Table**

**Table:** 

Subject area	*Physics, chemistry and pharmaceutics*
More specific subject area	*Molecular vibration on complexed local anesthetics*
Type of data	*Figures and table*
How data was acquired	*The Raman spectroscopy (RS) data were obtained on a MultiRAM FT spectrometer, Bruker, equipped with a Nd:YAG laser. The Fourier transformed infrared spectroscopy (FTIR) data was acquired on an* ATR Crystal spectrometer, Bruker*. The X-ray diffraction (XRD) data was collected on a D8 – Discover diffractometer, Bruker.*
Data format	*Raw and analysed data*
Experimental factors	*Powder samples*
Experimental features	*RS was collected between 200 and 3500 cm*^*-1*^*with an incident wavelength of 1064 nm and a laser powers of 250 mW. FTIR data were collected between 400 and 4000 cm*^*-1*^*with 500 scans for each sample. XRD data was collected with a Cu radiation source.*
Data source location	*Copenhagen, Denmark.*
Data accessibility	*Data are available in this article.*
Related research article	*Probing the dynamics of complexed local anesthetics via neutron scattering spectroscopy and DFT calculations*

**Value of the data**

• Relevant data on the characterization of local anesthetics RVC and BVC and the respective complexes.
• Data to be used on understanding molecular changes on local anesthetics after complexation in HP-β-CD.
• RS, FTIR and XRD data to be used as complementary information to several characterization techniques on pharmaceutical research.

## 1 Data

**Fig. 1:**
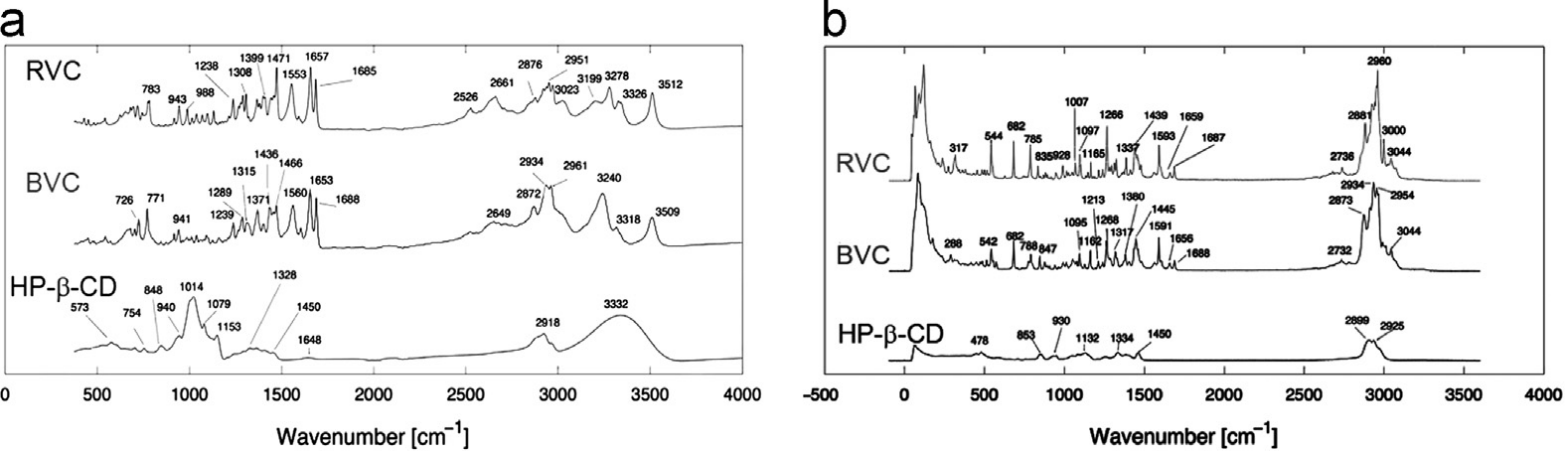
(a) FTIR and (b) Raman spectra of RVC, BVC and HP-β-CD. All data were collected at room temperature.

**Fig. 2:**
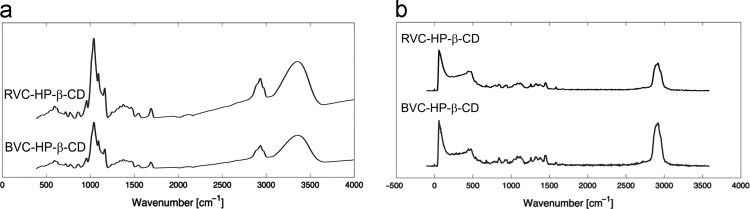
FTIR(a) and RS(b) data for RVC and BVC after complexation with HP-β-CD, thus RVC-HP-β-CD and BVC-HP-β-CD.

**Fig. 3:**
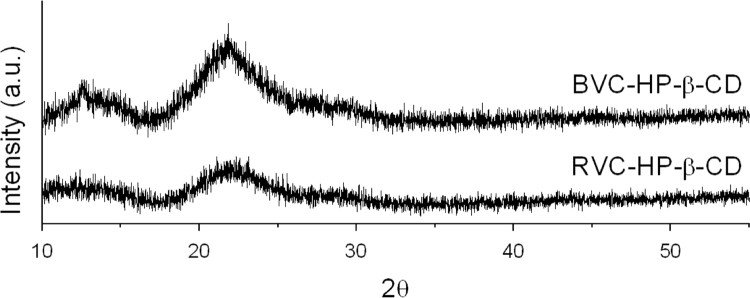
X-ray diffraction data for BVC after complexation with HP-β-CD (BVC-HP-β-CD) and for RVC after complexation with HP-β-CD (RVC-HP-β-CD). The data were collected with Cu radiation (λ = 1.54 Å). A baseline was subtracted from the data for background correction and BVC-HP-β-CD data was shifted for better visualization.

FTIR and Raman spectra for BVC, RVC and HP-β-CD are presented in Fig. 1 (a) and (b) to be used as complemmentary data for the neutron scattering analysis presented on reference [1]. Table 1 presents the modes assignment, based on references [2], [3], [4]. Fig. 2(a) presents FTIR data for BVC and RVC BVC after complexation with HP-β-CD, thus BVC-HP-β-CD and RVC-HP-β-CD. Fig. 2(b) shows the respective RS spectra. In Fig. 3, X-ray diffraction data is presented for BVC-HP-β-CD and RVC-HP-β-CD, i.e. RVC after complexation with HP-β-CD.

**Table 1:** Modes assignment for FTIR and RS for BVC, RVC and HP-β-CD.

**Sample**	**Frequencies (cm**^**-1**^**)**	**Modes Assignment**
***BVC and RVC***	• 3509 (BVC) • 3512 (RVC)	*O-H bond stretching*
	• *3240 and 3318 (BVC)* • *3199, 3278 and 3326 (RVC)*	*Stretching of hydrogen-bonded N-H group of the mono-substituted amides, O=C-N-H*
	*2960*	*CH*_*3*_*stretching*
	*2500 - 2700*	*Stretching of N-H-Cl*
	*1700 - 1600*	*C=C and C=O stretching*
	*1680 – 1630*	*Amide carbonyl stretching band* (□(*C=O*))
	*1550*	*Amide II vibration (C-N stretching vibrations together with N-H bending)*
	*1250*	*C-N-H stretch vibrations*
	*1470 - 1250*	*Information on the rings and the methyl (CH*_*3*_*) and methylene (CH*_*2*_*) groups*
	*1466, 1436 and 1471*	*CH*_*2*_*-bending*
	• *1371 (BVC)* • *1399 (RVC)*	*CH*_*3*_*bending*
	*1000 – 600*	*Bending of C-H groups located either in the rings or in the carbon groups*
	*Around 780*	*Adjacent CH wag modes*
		
**HP-β-CD**	3332	O-H bond vibration
	2928	C-H out of phase stretching
	1450 and 1328	C-H bending
	1153, 1079 and 1014	C-O stretching
	vibrations below 1000	Different types of bending of C-H bonds in the aromatic ring.

## 2 Experimental design, materials and methods

### 2.1 Materials

BVC hydrochloride monohydrate in the form of racemate (BVC.HCl, C_18_H_28_N_2_O.HCl.H_2_O) and RVC hydrochloride monohydrate (RVC.HCl, C_17_H_26_N_2_O·HCl·H_2_O) were donated by Cristália Prod. Quím. Farm. Ltda (Itapira, SP, Brazil). 2-hydroxypropyl−β−cyclodextrin, HP-β-CD, (Kleptose HP^®^) was obtained from Roquette Serv. Tech. Lab. (Lestrem, Cedex, France). Deionized water (Elga Maxima System, Elga, High Wycombe, UK) was used throughout the experiments. All other reagents were of analytical grade.

### 2.2 Sample preparation

Samples were prepared as described in [5]. Inclusion complexes were prepared by stirring equimolar amounts of the local anesthetics (racemate BVC.HCl and RVC.HCl) and HP-β-CD (1:1 M ratio) in deionized water at room temperature (25 ± 1) ^◦^C for 24 h. After completely dissolution and reaching equilibrium (4 h), the solution was freeze-dried (Labconco-freeze dry system/Freezone^®^ 4.5) and stored at -20 ^◦^C until further use.

### 2.3 Fourier Transformed Infrared Spectroscopy (FTIR)

FTIR spectra were collected to all samples at room temperature between 400 and 4000 cm^-1^, using an ATR Crystal from Bruker. For each sample 500 scans were carried out. A background measurement was collected at the beginning of the experiment, and the obtained signal was subsequently subtracted from all the other measurements.

### 2.4 Raman Scattering (RS)

RS between 200 and 3500 cm^-1^ were collected at room temperature using a MultiRAM FT-Raman spectrometer from Bruker equipped with a Nd:YAG laser. An incident wavelength of 1064 nm was used to measure the powder samples that were carefully mounted inside of glass vials. The powder samples were put in small glasses and a laser power of 250 mW was used to measure the HP-β-CD and the LA samples. Due to the lower density of the complex BVC-HP-β-CD, very thin pellets were made with the encapsulated drugs powders and a laser power of 500 mW was used. The data analysis was only qualitative in this experiment.

### 2.5 X-ray diffraction

BVC and RVC after complexation with HP-□-CD were investigated by X-ray powder diffraction (XPD) in a Brucker – D8 Discover diffractometer (Cu radiation – λ = 1.54 Å). The experiments were conduct with a 0.01° step, between 10° and 55°. A baseline was subtracted from the data for background correction and *BVC-HP-β-CD data was shifted for better visualization.*
