# Supplementary material for: Raman and Infrared spectroscopies and X-ray diffraction data on bupivacaine and ropivacaine complexed with 2-hydroxypropyl−β−cyclodextrin
Source: Data Brief. 2017 Sep 4;15:25–9. doi: 10.1016/j.dib.2017.08.053 (PMC5609877; doi:10.1016/j.dib.2017.08.053)
Supplement: Supplementary file 1 — Supplementary material [file mmc1.pdf]

UNIVERSITY OF COPENHAGEN

Dear Dr. Marina Irène Ostankovitch,

Editor, Data in Brief,

AUGUST, 9TH 2017

MURILLO LONGO MARTINS
NIELS BOHR INSTITUTE
(H.C. ØRSTED INSTITUTE, BLDG. D)
UNIVERSITY OF COPENHAGEN
UNIVERSITETSPARKEN 5
2100 COPENHAGEN DENMARK

On behalf of all authors, I hereby confirm that there are no known conflicts of interest associated with this publication and there has been no significant financial support for this work that could have influenced its outcome.

Sincerely yours,

Murillo L. Martins
Niels Bohr Institute - University of Copenhagen
Universitetsparken 5
2100 Copenhagen Denmark - murillolongo@gmail.com
